# Posttraumatic Stress Disorder and Adjustment Disorder in Lithuanian Healthcare in 2018–2020: A Nation-Wide Cohort Study of the Effects of COVID-19 Pandemic

**DOI:** 10.3390/healthcare9111422

**Published:** 2021-10-22

**Authors:** Evaldas Kazlauskas, Odeta Gelezelyte, Auguste Nomeikaite, Paulina Zelviene

**Affiliations:** Center for Psychotraumatology, Institute of Psychology, Vilnius University, M. K. Ciurlionio Str. 29, LT-03100 Vilnius, Lithuania; odeta.gelezelyte@fsf.vu.lt (O.G.); auguste.nomeikaite@fsf.vu.lt (A.N.); paulina.zelviene@fsf.vu.lt (P.Z.)

**Keywords:** PTSD, stress related, prevalence, healthcare

## Abstract

Multiple empirical studies have revealed significant pandemic effects of COVID-19 on mental health in various populations. This study aimed to analyze the incidences of posttraumatic stress disorder (PTSD) and adjustment disorder (AjD) in national healthcare in 2018–2020 in one of the European countries—Lithuania—and estimate the effect of the COVID-19 pandemic on PTSD and AjD incidences in 2020. The national healthcare registry was used for estimations of diagnosis of PTSD, AjD, and major depressive disorder (MD). The study revealed that stress-related disorders PTSD and AjD are diagnosed rarely, resulting in a considerable gap between the expected prevalence and incidences of these diagnoses in healthcare in Lithuania. Moreover, a significant decline in mental disorders incidence in healthcare in 2020, in comparison to 2018 and 2019, was found, revealing that the COVID-19 pandemic had a negative impact on access to healthcare services and increased barriers for mental disorders treatment. The study indicates that major developments in building up knowledge about the effects of trauma and life stressors on mental health are needed in Lithuania and other countries to increase awareness about stress-related disorders and improve care for trauma survivors, in particular in the context of the pandemics or other large-scale disasters.

## 1. Introduction

Posttraumatic stress disorder (PTSD) and adjustment disorder (AjD) are among the most often diagnosed mental disorders according to mental health professionals’ surveys [1,2]. Furthermore, according to epidemiological studies, PTSD prevalence rates are within 1–3% in the general population across Europe [3]. According to studies conducted in EU countries, the prevalence of ICD-10 adjustment disorder ranges from 0.9 to 1.4% in the general population in Germany [4], to as high as 16.5% in the general population in Lithuania [5]. Stress- and trauma-related disorders prevalence are much higher in high-risk groups [6]. However, even large-scale studies of trauma exposure and PTSD prevalence are often collected retrospectively; thus, the accuracy of these studies is limited [7].

Given the high prevalence of PTSD and AjD, it is not clear what is the treatment gap in PTSD or AjD treatments. The recognition and diagnosis of disorders specifically associated with stress, PTSD, or AjD, in healthcare, is crucial in delivering stress- and trauma-focused treatments for patients who were exposed to life stressors or traumatic experiences. Due to the considerable differences in healthcare systems worldwide and the diversity of health insurance, it is difficult to have a comprehensive outlook on the provision of PTSD and AjD treatments. Even in one region, such as Europe, there are large disparities in healthcare and trauma care in particular [8]. Health care utilization studies of the PTSD and AjD treatments are scarce; however, the few available studies reveal a low incidence of these mental disorders in healthcare across various countries.

A national survey of medical and social registries in Denmark revealed an average annual incidence of 4.7 per 100,000 population for PTSD, and an incidence of AjD of 85 per 100,000 population from 1995 to 2011 [9]. In a recent study in Korea, PTSD incidence in healthcare ranged from 4.2 to 8.3 per 100,000 of the population in the general population in 2011–2017 [10]. The study in Lithuania, an EU country with a population of about 2.7 million, reported a surprisingly low incidence of PTSD in Lithuanian healthcare in 2014–2015, with as low as <0.01% of the total population diagnosed with PTSD, identifying a treatment gap of more than 90% [11]. Meanwhile, AjD was diagnosed in 0.19–0.20% of the population in the same study [11]. The authors of the study argued that the lack of recognition of PTSD in healthcare might be associated with social factors and lack of training of healthcare professionals.

As PTSD and AjD are mental disorders that develop following adverse life events or stressors, a higher load of stressors can lead to increased prevalence of these disorders in a population. In the context of the COVID-19 pandemic, it is especially important to recognize PTSD and AjD among healthcare patients. It has been debated that the COVID-19 pandemic is associated with traumatizing experiences, such as being directly exposed to the virus, self-isolation, or loss of loved ones, which could have had an impact on mental health, including higher rates of PTSD and AjD [12,13]. Multiple studies have already found high rates of PTSD and AjD among healthcare workers, survivors of coronavirus infections, and the general population [14,15,16,17,18]. Studies show that women experience more distress during the COVID-19 pandemic [17], as well as more symptoms of depression, posttraumatic stress, and AjD [14,19]. Other risk factors for stress-related disorders during the COVID-19 pandemic include educational level, previous psychiatric history, and COVID-19 related worry. Furthermore, trauma-informed care is particularly important in the context of pandemics to help mitigate the effects of the pandemic on mental health [12]. Overall, considering findings from research, it is important to estimate the incidences of PTSD and AjD in healthcare in the context of the global pandemic, as it can help to understand how the healthcare system is coping with a potential increase in PTSD and AjD patients and can provide insights for healthcare provision planning and development in the current and future pandemics.

The first aim of this study was to evaluate the incidences of trauma- and stress-related disorders in Lithuanian national healthcare from 2018 to 2020. In addition, the study aimed to assess the impact of the COVID-19 pandemic on the rates of stress-related disorders, in particular PTSD and AjD in the year 2020, in comparison to the previous two years.

## 2. Materials and Methods

### 2.1. Data Source

Lithuania has national compulsory health insurance (PSD). All Lithuanian residents by law are contributing by monthly payments to the PSD, and healthcare services are covered by the PSD. The national registry of PSD SVEIDRA is a database of all services provided in healthcare institutions. Each individual visit to healthcare services is documented in the SVEIDRA database, and the database has millions of entries annually. Data for this study were extracted from SVEIDRA by the Health Information Center of Hygiene Institute, which is a governmental institution functioning under the Ministry of Health of the Lithuanian Republic. The Health Information Center is the key institution in the country responsible for the management of public health registries and monitoring the health of the Lithuanian population.

### 2.2. Data Extraction

The 10th edition of International Classification of Diseases—Australian Modification (ICD-10-AM) is used in the national healthcare registry SVEIDRA for coding the diseases. For the purpose of this study, aggregated national data on the incidences of diagnosis were extracted without identifying patient information. All individuals who had been diagnosed at least once in 2018–2020 with the following trauma- and stress-related mental disorders diagnoses were included in the analysis: PTSD (ICD-10 code F43.1), adjustment disorder (AjD; F43.2). Additionally, we included major depressive disorder (MD, F32, F33) in the analysis. Data were extracted for the total population, as well as gender-specific population.

### 2.3. Data Analysis

For estimation of the incidences of mental disorders in healthcare, we used an annual population size from the official Lithuanian national statistics registry [20]. Incidences of PTSD, AjD, and MD diagnosis per 100,000 of the population were computed. Chi-squared analyses were used to test for differences between men and women in the prevalence of these disorders. Fisher’s exact test was used to assess the effect of the COVID-19 pandemic on the prevalence of mental disorders by comparing the occurrence of diagnosed mental disorders in national healthcare in 2020, which was mostly affected by the COVID-19, vs. average cases of mental disorders in the previous two years (2018 and 2019) while controlling the population size changes. The total population size in 2018 was 2.81 million, while in 2019, it was 2.79 million, and in 2020, it was 2.79 million [20].

### 2.4. The Situation of COVID-19 in Lithuania in 2020

From 16 March to 17 June 2020, the first lockdown was implemented in Lithuania [21]. During this period, restrictions, such as mandatory 14-day isolation for arrivals, mandatory face masks, and restrictions on cross-border movement were set. From 21 October 2021, the government introduced colour zones throughout Lithuania—green zones indicated less strict restrictions, while red zones indicated strict restrictions were enforced. However, due to the growing numbers of COVID-19 cases, the government announced a second national lockdown on 7 November, and it lasted until 1 July 2021. During this second lockdown period, Lithuania had restrictions such as limited contact outside the household and restrictions on movement between municipalities. A total of 141,955 cases of COVID-19 were diagnosed in Lithuania in 2020 starting since March 2020, with 2220 diagnosed COVID-19-related deaths in 2020.

## 3. Results

### 3.1. Incidents of Trauma- and Stress-Related Disorders in Healthcare

Data on PTSD, AjD, and MD in Lithuanian healthcare are presented in Table 1. The total number of diagnosed cases of PTSD in national healthcare ranged between 351 and 437 cases, with incidences per 100,000 of population ranging from 12.56 to15.64, which is 0.01–0.02% of the total population. AjD was diagnosed in healthcare from 6611 to 7390 times (0.24–0.26% of the total population), with a 236.61–264.48 incidence rate in the years 2018–2020. Over the analyzed time, MD cases ranged from 43,009 to 46,820 (1.54–1.67% of the population) and 1539.28–1668.93 incidence rates in national healthcare.

We found a significant gender effect for all three mental disorders analyzed, with significantly higher incidence rates among females in comparison to males (See Table 1). The gender ratio for the diagnosis of PTSD and MD was approximately 1:3, and 1:4, respectively. AjD was more equally distributed among female and male patients, with around 35% of males diagnosed with AjD.

### 3.2. The Role of the COVID-19 Pandemic

To assess the role of the COVID-19 pandemic, we compared incidents of mental disorders in 2020 with the averaged diagnosis numbers in 2018–2019 in the same registry. Analysis revealed a significant decline in PTSD in national healthcare in 2020, compared with 2018–2019, (Fisher’s exact test, *p* = 0.012). Furthermore, in the case of the adjustment disorder, we also found a significant decline in 2020, (Fisher’s exact test, *p* < 0.001). The incidence of MD in the national healthcare sector in 2020 in comparison to the 2018–2019 average (Fisher’s exact test, *p* < 0.001) was also significant.

## 4. Discussion

The study revealed in general low incidences of PTSD and AjD in national healthcare in Lithuania. In line with a previous study, we found that national healthcare in Lithuania is struggling in diagnosing trauma- and stress-related disorders [11]. PTSD and adjustment disorders are diagnosed scarcely, and in contrast to the epidemiological large-scale studies, there is a considerable gap between the expected and diagnosed PTSD or AjD. In this regard, major depressive disorder is diagnosed significantly more often, more than 100 times a year, in comparison to PTSD, and more than about 7 times, in comparison to AjD in Lithuanian healthcare. The treatment gap for MD is much lower in contrast to AjD or PTSD.

Our study findings are similar to other registry or healthcare utilization studies, which found a low incidence of PTSD and AjD in healthcare registries and healthcare insurance databases. We found higher incidence rates in Lithuania in comparison to Denmark [9] or Korea [10]; however, these studies have been conducted on registry data from 1999 to 2017, and therefore, more recent data analysis in these countries could reveal different outcomes. Furthermore, we found significantly higher gender effects in our sample. A high female proportion was found in PTSD and MD incidences in Lithuanian healthcare. These results are in line with other studies that have found that women in the European region have a higher prevalence of mental health disorders than men [22]. This raises concerns about cultural factors that may make women more likely to suffer from the negative effects of life stress factors on their emotional well-being. On the other hand, research shows that women are more likely to seek mental help in healthcare [23]. This may be due to barriers for males to enter mental health services, potentially due to the stigma of mental disorders in Lithuanian society. Another possible explanation is that men are more likely to seek self-medicating ways via excessive alcohol use [24]. Research in Lithuania shows that men’s mortality, similar to other post-Soviet countries, is associated with high alcohol consumption [25], and men are as much as three times more likely to have harmful alcohol consumption habits than women [26].

The year 2020 was marked by the global pandemic and its repercussions. In Lithuania, as in other European countries, the first COVID-19 patients were diagnosed in the first quarter of 2020, and soon, the strict national lockdown was imposed. Contrary to the empirical studies and expert opinion on the traumatizing nature of the COVID-19 pandemic and indications that it can be associated with the high prevalence of PTSD [15] or AjD [13], we found no increase in PTSD or AjD incidence in healthcare in 2020. Moreover, we found a decline in mental disorders, including MD, in healthcare in 2020. There might be several explanations for these findings. Due to the lockdown, access to non-emergency healthcare services, including mental health services, was restricted. There were no routine remote online services in healthcare, as these were predominantly face-to-face services prior to the COVID-19 pandemic. It took time to adjust healthcare to the delivery of services online, both diagnostic and treatment, including those of mental health. Furthermore, disorders such as adjustment disorders or PTSD usually develop after some time, and thus, we may have more adjustment disorders later on in 2021 or 2022. Another explanation for these results would be that the measures of restriction of mobility for some individuals actually acted as protective factors with respect to distress, as they strengthened close emotional ties and consequently the processes of meaning making, which, in turn, reduce the negative impact of events on distress [27,28,29].

The study has several limitations. As this was a healthcare registry study, our data extraction was limited to the data available from the registry. We could not identify if PTSD or AjD cases diagnosed in 2020 were associated with the COVID-19 pandemic-related trauma or stressor exposure. In addition, we could include only data from the official healthcare registry. The current legislation in Lithuania allows diagnostic decisions only for medical doctors, and other specialists, such as psychologists, are not allowed to diagnose. As some psychological counseling and psychotherapy services are provided in private practice or in social or educational settings outside of healthcare institutions, some individuals with mental disorders may receive these services without the inclusion of data in the healthcare registry. However, only licensed healthcare institutions can officially diagnose disorders in Lithuania; therefore, these other services—even if they have clients with PTSD or AjD—do not diagnose them. Furthermore, we reported incidents of only diagnosed cases; it is possible that some patients who seek treatment in healthcare for their stress-related disorders are unrecognized or misdiagnosed with other mental disorders, and thus were not included in our study. Further, we included all individuals diagnosed with PTSD, AjD, and MD annually even if a patient was diagnosed with a stress-related disorder or MD as a comorbid disorder. However, we did not analyze PTSD and AjD comorbidities due to the limitations of our study. It would be interesting to investigate how stress-related disorders overlap with other medical conditions or mental disorders. Finally, we did not explore which care providers diagnose PTSD and AjD more often and whether they are primary care or specialized facilities, such as mental health hospitals; this could be further explored in future studies.

Even in light of these limitations, the most important strength of the study is a large-scale national registry study design that enables the estimation of stress-related disorders diagnostic situations in national healthcare in one of the European Union countries. The findings of our study reveal that trauma- and stress-related disorders are not diagnosed in routine clinical care in Lithuania, which is a troubling finding due to the increase in the rates of mental disorders associated with the COVID-19 pandemic worldwide. However, we can expect that this is not a country-specific finding but rather a tendency in the region and, supposedly, in many other countries around the globe. Undetected and untreated PTSD and AjD can result in other mental disorders, such as substance abuse or social problems. The study could not reveal the reasons behind the lack of recognition of PTSD and AjD among healthcare professionals; however, it is quite clear that it may be associated with a lack of knowledge about the effects of adversities on mental health and limited capacity of mental health services to adapt to the large-scale disasters, such as the global pandemic. Additionally, the pandemic revealed that novel eHealth solutions and digital diagnostic tools, as well as online provision of treatment, are important.

## 5. Conclusions

Our correlational study revealed a large gap between the expected and observed prevalence of PTSD and AjD based on the incidences in national healthcare in Lithuania. Moreover, we found that the incidences of PTSD, AjD, and MD decreased in 2020, in comparison to the previous two years, indicating that the COVID-19 pandemic had a significant effect on mental health services and increased the burden of mental disorders due to restricted access to healthcare. Further studies in other countries are needed to estimate the worldwide impact of the COVID-19 pandemic on the prevalence of mental disorders in healthcare.

## Figures and Tables

**Table 1:** Cases of stress-related disorders and depression in Lithuanian national healthcare in 2018–2020.

Mental Disorder	Year	Total		Male			Female			Χ^2^
		*n*	Rate	*n*	Rate	Residual	*n*	Rate	Residual	
Posttraumatic stress disorder (F43.1)	2018	408	14.53	97	7.48	−90.7	311	20.57	90.7	81.14 ***
	2019	437	15.64	106	8.18	−95.0	331	22.09	95.0	83.18 ***
	2020	351	12.56	81	6.21	−84.0	270	18.12	84.0	80.64 ***
Adjustment disorder (F43.2)	2018	7123	253.59	2568	197.95	−708.6	4555	301.33	708.6	283.77 ***
	2019	7390	264.48	2729	210.64	−670.4	4661	311.03	670.4	244.83 ***
	2020	6611	236.61	2350	180.17	−757.2	4261	286.02	757.2	348.13 ***
Major depressive disorder (F32, F33)	2018	46,820	1666.84	8996	693.44	−12,541.2	37,824	2502.24	12,541.2	13,523.69 ***
	2019	46,633	1668.93	9008	695.28	−12,443.2	37,625	2510.69	12,443.2	13,366.50 ***
	2020	43,009	1539.28	8209	629.35	−12,005.2	34,800	2335.98	12,005.2	13,452.65 ***

*Note*. *** *p* < 0.001. The incidence rate (Rate) is calculated per 100,000 of population.

## Data Availability

Data from the national registry were extracted for the study. No empirical data were collected in the study, and thus, data are not available for sharing.
